# Diagnosis delay of breast cancer and its associated factors in Malaysian women

**DOI:** 10.1186/1471-2407-11-141

**Published:** 2011-04-17

**Authors:** Bachok Norsa'adah, Krishna G Rampal, Mohd A Rahmah, Nyi N Naing, Biswa M Biswal

**Affiliations:** 1 Unit of Biostatistics and Research Methodology, School of Medical Sciences, Universiti Sains Malaysia, 16150 Kubang Kerian, Kelantan, Malaysia; 2 Department of Community Health, Faculty of Medicine, Universiti Kebangsaan Malaysia, 56000 Cheras, Kuala Lumpur, Malaysia; 3 Department of Medical Nuclear, Radiotherapy and Oncology, School of Medical Sciences, Universiti Sains Malaysia, 16150 Kubang Kerian, Kelantan, Malaysia

**Keywords:** breast cancer, diagnosis delay, consultation delay, presentation delay, patient delay

## Abstract

**Background:**

Breast cancer is the leading cause of cancer mortality among women in Malaysia. Delayed diagnosis is preventable and has major effects on patients' prognosis and survival. The objectives of our study were to identify the magnitude of delayed diagnosis and its associated factors in women with breast cancer in Malaysia.

**Methods:**

This study had a cross-sectional design. Respondents had histologically confirmed breast cancer and were registered at five medical centres between 2005 and 2007. All breast cancer patients who attended hospital clinics at the East Coast were included. Patients at Kuala Lumpur hospitals were selected by systematic sampling. A standardised questionnaire was developed to interview respondents. We measured the time from the first recognition of symptoms to the first general practitioners' consultation and to the histological diagnosis of breast cancer. Diagnosis delay was defined when there was more than 6 months from the recognition of symptoms to the histological diagnosis. Multiple logistic regression was used for analysis.

**Results:**

In total, 328 respondents were included. The mean (standard deviation) age was 47.9 (9.4) years. Most respondents were of Malay ethnicity, were married housewives with a median family income of RM1500 a month. Most respondents had ductal carcinoma (89.3%) and the stage distribution was as follows: 5.2% stage I, 38.7% stage II, 44.8% stage III and 11.3% stage IV. The median time to consultation was 2 months and the median time to diagnosis was 5.5 months. The frequency of diagnosis delay of more than 3 months was 72.6% and delay of more than 6 months occurred in 45.5% of the cases. The factors associated with diagnosis delay included the use of alternative therapy (odds ratio (OR) 1.77; 95% confidence interval (CI): 1.06, 2.94), breast ulcer (OR 5.71; 95% CI: 1.59, 20.47), palpable axillary lymph nodes (OR 2.19; 95% CI: 1.23, 3.90), false-negative diagnostic test (OR 5.32; 95% CI: 2.32, 12.21), non-cancer interpretation (OR 1.68; 95% CI: 1.01, 2.78) and negative attitude toward treatment (OR 2.09; 95% CI: 1.15, 3.82).

**Conclusions:**

Delays in consultation and diagnosis are serious problems in Malaysia. Diagnosis delay was influenced by complex interactions between many factors. Breast awareness and education are required to promote early detection, diagnosis and treatment before the tumours enlarge and metastasis.

## Background

There were 3525 cases of female breast cancer that were registered in the National Cancer Registry Malaysia in 2006, accounting for 16.5% of all cancer cases registered that year [1]. The overall age-standardized rate was 39.3 per 100,000 populations [1]. The cancer incidence in Malaysia is expected to increase because of increasing life expectancy, better socio-economic status and changes in lifestyle. Patients with breast cancer in Malaysia commonly present with advanced disease. The Kelantan Cancer Registry reported that 19.0% of patients presented in stage I, 25.5% in stage II, 20.7% in stage III and 34.9% in stage IV [2]. It was previously reported that the 5-year survival rate in Kuala Lumpur was 59.1% [3], whereas this rate was 25.8% in Kelantan [4].

The delay in the presentation and detection of patients with breast cancer is partially responsible for the advanced stage at presentation and low survival rates in Malaysia. Early detection of cancer is important because delay is preventable and earlier treatment can lead to improved survival. Late detection has been associated with larger tumour size, increased involvement of the lymph nodes and organ metastases [5], and negative implications on cost and choice of treatment. A study in Thailand reported that patients with stage III disease had a median delay of presentation of 2 months compared to those in stage IV (7 months) [6]. In addition to lower survival rates, patients with delays of 12 weeks or longer had a significantly higher probability of local cancer spread or distant metastases compared to those with shorter delays [7].

Studies in developed countries reported that the median time to consultation was 14-61 days [8-10]. A delay of more than 3 months prior to physician consultation occurred in 14-53% of cases [8-11]. Low socio-economic status, minority ethnicity and young age were associated with a longer duration of symptoms [12]. Another study found that patient delay was associated with older age, lighter symptoms, not informing anyone, a negative attitude toward medical practitioners and fear of treatment [13]. Failure of medical practitioners to act on clinical findings and false-negative mammogram and fine needle aspiration cytology (FNAC) were the main factors for system delay [14,15]. Lack of knowledge regarding risk factors, individual own risk and the variability of symptoms of breast cancer, was related to patient delay [16].

This study was conducted to identify the magnitude of the delay in breast cancer diagnosis and the factors associated with this delay. To date, most of the research on this topic has been conducted in developed countries and among minorities, and very few studies have been conducted in less developed countries. No studies on this subject in Malaysia have been published. Research on this topic is important for clinicians to have a better understanding of how to manage patients and so that policy makers can implement strategies and activities to prevent delay in breast cancer diagnosis.

## Methods

This study had a cross-sectional design. The respondents were women who were diagnosed with primary breast cancer by histo-pathological examination between 2005 and 2007. We excluded patients with cognitive problems, recurrent cancer and incomplete medical records. Respondents were from three referral medical centres in the East Coast of Malaysia and two government hospitals in Kuala Lumpur. All patients who attended surgical, oncology and radiotherapy clinics at the East Coast hospitals during our study period were included. Systematic sampling was conducted on every fourth eligible patient at Kuala Lumpur hospitals.

Face-to-face interviews were conducted using standardized questionnaires. The questionnaire was developed in the Malay language based on expert discussions and a literature review. It was pre-tested for face and content validity and reliability, which were satisfactory (Cronbach's Alpha 0.63-0.92). The content included socio-demography, medical and obstetric history, the date of all the chronological events (i.e., first recognition of symptoms, first consultation, referral, first hospital appointment, first meeting with the surgeon and oncologist, diagnostic tests and when the results were known) and the use of alternative therapy. We considered the symptoms' interpretation process spanned from the point at which the women started to notice abnormalities until a diagnosis was made [17]. An agreement was decided after discussion with the respondents when there were conflicting dates of events. The respondents were reminded of events in the calendar year, such as festival celebrations, Independence Day, school holidays or birth dates, to help them remember important dates relative to their medical history.

We also included yes-no questions on the interpretation of symptoms, knowledge about presenting symptoms, aetiology and metastatic organs, beliefs about breast cancer and treatment, fear, denial, barriers, healthcare services, husband support, attitude on medical care and treatment, and health care practices. A 'yes' response for positive questions was given a score of one and a 'no' response was given a zero. The scoring was reversed for the negative questions. All scores for each group were totalled and then categorised using the median of the total score. We also collected data from the medical records including clinical presentation, diagnostic tests, histo-pathological reports and treatment information.

The Psycho-physiological Comparison Theory developed by Andersen, Cacioppo and Roberts was taken into consideration when we formulated the operational definition of delay [18]. Consultation time was the time taken to visit the first general practitioner after the recognition of symptoms. The time to diagnosis was measured from the date of the recognition of symptoms to the date of final diagnosis based on histo-pathological examination of FNAC, trucut or excision biopsy. Breast cancer was staged according to the 6^th ^edition of Cancer Staging Manual published by American Cancer Joint Committee on Cancer [19]. A family history of breast cancer was defined as having a first-degree relative, i.e. sister, mother or daughter who had breast cancer. The respondents were questioned about previous use of oral contraceptives, hormone replacement therapy or alternative therapy if these therapies were taken regularly for at least one month. Complementary alternative therapy was defined as any therapy using methods and products not included in conventional modern medicine. Chronic diseases assessed in this study included hypertension, diabetes, heart diseases, asthma and other diseases that require lifelong monitoring. Misdiagnosis occurred when there was a false-negative mammogram or FNAC.

The research had been approved by the Research and Ethical Committee from all the respective institutions, with the reference numbers: UKM1.5.3.5/244/SPP2, HKL/98/AM.882, USMKK/PKK/JK EP(M)-191 USM, Bil(43)HRPZ ll.71/20 Jld.8, HSNZ.KT.100-22/15(27) and (4)KKM/NIHSEC/08/0804/P07-13. Respondents were explained about the research and asked for consent prior the interview. All information was confidential and individual data had no identification of the respondents.

### Statistical analyses

Data were analysed using SPSS for Windows (version 12.0.1, SPSS Inc., Chicago, IL, USA). Continuous data were summarised as mean (standard deviation (SD)) or median (interquartile range (IQR)) depending upon the normality of distribution, whereas categorical data were presented as frequency (percentage (%)). We divided the diagnosis time into a binary outcome, i.e., delay and non-delay, by using a six-month cut-off point. Six months was chosen instead of three month to allow balance number of respondents in each category. Multiple logistic regression was used to identify the factors associated with diagnosis delay. A stepwise backward selection procedure was used when selecting significant variables in the model. The interaction terms and multi-collinearity problem of the final model were checked. The final model was tested for fitness using the Hosmer-Lemeshow goodness of fit test. Results were presented as the crude and adjusted odd ratios (OR), 95% confidence interval (CI) and *p *value. The *p *value <0.05 was considered to indicate statistical significance.

## Results

### Background of the respondents

In total, 328 respondents were included in the final analysis. Table 1 shows the socio-demographic characteristics of the respondents. The mean age was 47.9 years (SD 9.4). The majority of the respondents were Malays, who were married housewives with at least a high school education and a median family income of RM1500 a month. Table 2 shows the medical history of the respondents. Only 8.2% had a family history of breast cancer, 12.2% had previous benign breast diseases and 31.4% had comorbid chronic diseases. Twelve percent did not have any children and 30.5% were post-menopausal. Among the 100 post-menopausal women, only 16.0% had ever taken hormone replacement therapy.

**Table 1:** Socio-demographic characteristics of the respondents

Socio-demography	Frequency (%) N = 328	Mean (SD)
Age at diagnosis (year)		47.9 (9.4)
40 & less	56 (17.1)	
>40	272 (82.9)	
Ethnicity		
Malay	262 (79.9)	
Chinese	46 (14.0)	
Indian	14 (4.3)	
Others	6 (1.8)	
Education level		
None	39 (11.9)	
Primary school	52 (15.9)	
Middle school	37 (11.2)	
High school	128 (39.0)	
Upper high school	13 (4.0)	
Diploma	28 (8.5)	
Degree	31 (9.5)	
Years of education		9.8 (4.6)
Monthly family income (RM)		1500 (2338)*
Occupation		
Housewife	180 (54.9)	
Government servant	78 (23.8)	
Private sector	44 (13.3)	
Self-employed	13 (4.0)	
Unemployed	13 (4.0)	
Marital status		
Married	260 (79.3)	
Widow	39 (11.8)	
Single	18 (5.5)	
Divorce	11 (3.4)	

*median (IQR), US$1~Ringgit Malaysia (RM) 3.10

**Table 2:** Medical history of the respondents

Medical history	Frequency (%)
Family history of breast cancer	27 (8.2)
History of benign breast disease	40 (12.2)
Parity status	
Nulliparous	40 (12.2)
Parous	288 (87.8)
Co-morbid chronic disease	103 (31.4)
Oral contraceptive pills	123 (37.5)
Menopausal status	
Pre	228 (69.5)
Post	100 (30.5)
Hormone replacement therapy (n = 100)	16 (16.0)

Table 3 shows the clinical characteristics of the respondents. Most cases occurred in the right breast (54.0%) and 2.7% had a second breast cancer in the contralateral breast. The appearance of a lump was the most common first symptom. Most (97.6%) had a breast lump at diagnosis. Less than 9% had at least one symptom of systemic involvement. Table 4 shows the histo-pathological findings and treatment of the respondents. In total, 12.9% of the respondents had benign FNAC and 8.8% had false-negative mammograms. Most respondents had invasive ductal carcinoma (89.3%). No respondents had carcinoma in situ. A total of 37.5% of the respondents had grade 3 disease, 44.8% had stage III and 11.3% had stage IV. Furthermore, 48.2% had oestrogen-receptor-positive, 50.3% had progesterone-receptor-positive and 34.8% had C-erb B_2_-positive tumours. Most underwent mastectomy (73.8%), 19.2% had breast conserving surgery and 86.3% and 78.4% completed chemotherapy and radiotherapy, respectively. There were 48 (14.6%) respondents who initially refused treatment and 15 (4.6%) respondents refused chemotherapy. In total, 140 (42.7%) respondents took alternative therapy and 48 (14.6%) missed at least one appointment without acceptable reasons. Out of 140 respondents who took alternative therapy, 67.1% drank enchanted water or applied rice flour locally to the breast lump.

**Table 3:** Clinical presentations of the respondents

Clinical characteristics	Frequency (%)
Location of tumour on right	177 (54.0)
Second breast cancer on contralateral breast	9 (2.7)
First symptom	
Breast lump	288 (87.8)
Nipple problems	12 (3.6)
Breast pain	10 (3.1)
Changes of breast shape	9 (2.8)
No symptom	4 (1.2)
Others	5 (1.5)
Symptom/sign during diagnosis (not exclusive)	
Breast lump	320 (97.6)
Nipple retraction	63 (19.2)
Pain at breast	47 (14.3)
*Peau de orange*	36 (11.0)
Breast ulcer	28 (8.5)
Gross swelling	25 (7.6)
Nipple discharge	22 (6.7)
Fungating	19 (5.8)
Breast dimpling	7 (2.1)
Arm oedema	5 (1.5)
Axillary lymph nodes	87 (26.5)
Supraclavicular lymph nodes	14 (4.3)
Systemic symptom/sign during diagnosis (not exclusive)	
Loss of weight	28 (8.5)
Loss of appetite	20 (6.1)
Bony pain	7 (2.1)
Cachexia	4 (1.2)
Cough	2 (0.6)
Bone fracture	1 (0.3)
Short of breath	1 (0.3)

**Table 4:** Histo-pathological findings and treatment of the respondents

	Frequency (%)
Benign Fine Needle Aspiration Cytology (FNAC) (n = 241)	31 (12.9)
False-negative mammogram (n = 136)	12 (8.8)
Invasive ductal carcinoma	293 (89.3)
Stage of breast cancer	
I	17 (5.2)
II	127 (38.7)
III	147 (44.8)
IV	37 (11.3)
Bloom Richardson Grade	
1	55 (16.8)
2	150 (45.7)
3	123 (37.5)
Estrogens receptor	
Positive	158 (48.2)
Negative	149 (45.4)
Not known	21 (6.4)
Progesterone receptor	
Positive	165 (50.3)
Negative	142 (43.3)
Not known	21 (6.4)
C-erb B_2_	
Positive	114 (34.8)
Negative	141 (43.0)
Not known	73 (22.2)

### The magnitude of diagnosis delay

**Figure 1:**
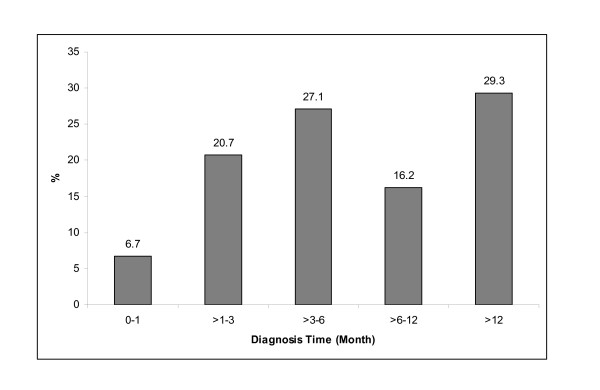
**Diagnosis time of 328 respondents with breast cancer**.

The times to consultation and diagnosis showed skewed distributions to the right. The range of consultation time was 0-11 years and the median was 2 months. Approximately 33.2% of respondents had a medical consultation within one month after detecting symptoms and 43.3% delayed the consultation by more than 3 months. The range of diagnosis time was 0-16 years and the median was 5.5 months. Figure 1 shows the diagnosis time according to time categories. The frequency of diagnosis delay of more than 3 months was 72.6% and the frequency of diagnosis delay of more than 6 months was 45.5%.

### Factors associated with diagnosis delay

Variables with *p *value <0.25 in the univariable logistic regression were included in the selection of variables in the multiple logistic regression modelling. The variables included the following: locality, ethnicity, years of education, employment, family income, breastfeeding, use of alternative therapy, first symptom, first doctor's action, self-detection of symptoms, nipple retraction, breast ulcer, gross breast swelling, fungating mass, *peau de orange*, loss of weight, palpable axillary lymph node, palpable supraclavicular lymph node, false-negative diagnostic test, defaulter, stage of disease, cancer interpretation, perceived barrier, attitude of medical consultation, social support and attitude toward treatment. The final multiple logistic regression analysis model is shown in table 5. The factors significantly associated with diagnosis delay were the use of alternative therapy (OR 1.77; 95% CI: 1.06, 2.94), breast ulcer (OR 5.71; 95% CI: 1.59, 20.47), palpable axillary lymph nodes (OR 2.19; 95% CI: 1.23, 3.90), false-negative diagnostic test (OR 5.32; 95% CI: 2.32, 12.21), non-cancer interpretation (OR 1.68; 95% CI: 1.01, 2.78) and negative attitude toward treatment (OR 2.09; 95% CI: 1.15, 3.82). The result of the Hosmer-Lemeshow goodness of fit showed that the selected model had a good fit.

**Table 5:** Factors associated with diagnosis delay of breast cancer in Malaysian women

Associated factors		Frequency (%)		Crude Odd Ratio^a^ (95% CI)	Adjusted Odd Ratio^b^ (95% CI)	*P* value^b^
					
		Non-delay N = 179	Delay N = 149			
Alternative therapy	No	122 (68.2)	66 (44.3)	1.00	1.00	0.029
	Yes	57 (31.8)	83 (55.7)	1.69 (1.33, 2.1)	1.77 (1.06, 2.94)	
Breast ulcer	No	176 (98.3)	124 (83.2)	1.00	1.00	0.008
	Yes	3 (1.7)	25 (16.8)	2.16 (1.79, 2.60)	5.71 (1.59, 20.47)	
Palpable axillary lymph nodes	No	148 (82.7)	93 (62.4)	1.00	1.00	0.008
	Yes	31 (17.3)	56 (37.6)	1.67 (1.33, 2.08)	2.19 (1.23, 3.90)	
False-negative diagnostic test	No	169 (94.4)	122 (81.9)	1.00	1.00	<0.001
	Yes	10 (5.6)	27 (18.1)	1.74 (1.37, 2.21)	5.32 (2.32, 12.21)	
Interpret symptom as cancer	Yes	87 (48.6)	56 (37.6)	1.00	1.00	0.044
	No	92 (51.4)	93 (62.4)	1.57 (1.01, 2.44)	1.68 (1.01, 2.78)	
Attitude toward treatment	Positive	151 (84.4)	109 (73.2)	1.00	1.00	0.016
	Negative	28 (15.6)	40 (26.8)	1.98 (1.15, 3.40)	2.09 (1.15, 3.82)	

^a^Univariable logistic regression ^b^Multiple logistic regression

## Discussion

Our study found that consultation and diagnosis delay among breast cancer patients are very serious problems in Malaysia. In the present study, the median times before consulting a medical practitioner and before diagnosis were longer [6,8,9,20], and the prevalence of delay was higher, than in other studies in developed and developing countries [5,6,9-11].

Our study found that the clinical presentation was associated with a delay, which is supported by the literature [21]. Our study also found that patients who presented with breast ulcer and palpable axillary lymph nodes had significantly delayed diagnoses. The delay would eventually cause breast cancer symptoms to worsen. The first symptoms of breast cancer are usually not debilitating and can be ignored until the occurrence of new symptoms or worsening of symptoms. Patients with aggressive types of breast cancer usually had progressive symptoms in a short time, causing them to have a high index of cancer suspicion and seek early consultation and diagnosis. Patients who had a breast lump had earlier consultations than those with nipple discharge [9] or pain [22] or non-lump symptoms [23].

False-negative FNAC or mammogram contributed to the delayed diagnosis of breast cancer in our study. Only 241 (73.5%) of respondents had FNAC and only 51.2% had positive results of breast cancer and 12.9% reported as benign. The false-negative FNAC rate in our study was higher than that reported in other studies, with only 1.0-1.9% [15,24,25]. Most of the errors occurred during the performance of the FNAC procedure or the interpretation of the pathological report.

Only 136 (41.5%) of respondents in our study had mammogram and 8.8% of those were false negative, which is a higher rate than the previous study of only 3% [15]. The acceptable false negative rate of mammogram was 10-15% [26]. The failure of mammograms to detect breast cancer was related to the limitations of the film screen, poor radiographic technique, exceptional tumour characteristics and error in interpreting the film [26]. In Malaysia, mammograms are not used for screening except for high-risk women who have had previous breast cancer or a strong family history of breast cancer. It is not used for screening because of the high cost and limited expertise in Malaysia. In Malaysia, there are clinical practice guidelines for breast cancer management, but the use and implementation of the guidelines are unknown. More widespread implementation of these guidelines may increase the quality of care for breast cancer patients and shorten the diagnosis delay.

In our study, 42.7% of the respondents had taken alternative therapy, compared to 14.8-73.1% in Europe [27]. Most patients took alternative therapy as a way to avoid surgery or when they perceived modern medicine would not cure the disease, when the prognosis was fatal, when the disease caused suffering or in cases of chronic disease. Some patients believed that there were no effective treatments for breast cancer or that traditional medication is more effective than modern medicine. While pursuing alternative treatments, most patients experienced worsening symptoms, which led to them eventually presenting at a more advanced stage. A systematic review reported that people practiced complementary alternative medicine because of its benefits and because they wanted to be in control of their treatment, had strong beliefs and used it as the last hope [27]. Some patients also have less trust in modern medicine because they had bad previous experiences or felt that the system was not as friendly as traditional healers or shaman. Complementary alternative therapy is also easily available and affordable.

Patients' interpretation of their symptoms as a sign of cancer had an important influence on whether they sought medical help immediately [28]. The evaluation of breast symptoms is based on the pre-existing knowledge, experience, self-education and observation of individuals [18]. Knowledge regarding the variation of symptoms in breast cancer enables patients to interpret the symptoms correctly and influences their assessment of symptoms as well as their decision to seek medical attention [13].

Patients are more inclined to attribute new symptoms to less serious conditions instead to a life threatening disease [18]. Patient delay has been reported for patients who assumed that symptoms were benign [23] and would disappear without intervention. Perception of the seriousness of a symptom is dependent upon the first symptom and how fast the symptom changes and multiplies. Most of breast cancer symptoms are mild, not specific, unclear, confusing, do not require urgent attention and can be ignored temporarily.

Our study found that a negative perception of breast cancer treatment prevented patients from receiving early diagnoses, similar to a study in Nigeria [29]. Negative information, such as the side-effects of chemotherapy, led to fear and caused some patients to refuse treatment. Some believed that the effects of chemotherapy were worse than breast cancer itself. Another negative perception of breast cancer treatment was related to the traditional woman's role in the family of taking care of children and the husband. Some believed that treatment would disrupt and burden their family because they could not perform their usual roles and might even have to rely on others to care for them. Because the women could not take care of the family and the husbands usually had difficulty in taking over the roles of the women, the husband might choose to separate or take another wife. Fear of divorce or the husband remarrying could lead some women to decide not to get their symptoms diagnosed if they suspected breast cancer. Some patients also believed that breast cancer could not be cured [17], so there was no point of having it diagnosed and treated. Diagnosis delay was also related to a belief that mastectomy causes disfigurement and disability [28].

The strength of our study lies in the fact that it was conducted at five large medical centres in Malaysia. One of the medical centres on the East Coast was the only centre that offered oncology and radiotherapy services in that area. The chances of patients receiving treatment elsewhere were minimal because most patients could not afford private services. However, a multicenter study leads to a considerable variation in the management of breast cancer patients because medical practitioners from different hospitals have different preferences.

We collected relevant dates based on the detailed approach described by Andersen, Cacioppo and Roberts [18]. Dates of events were collected via interviews with support from the medical records. Previous report suggests that the collection of actual date is more accurate than asking about the duration of time [30]. Our study used face-to-face interviews, in contrast to most other studies that used the existing medical records, postal or telephone interviews that had low response rates. Researchers have no control over the data if the medical records are used. There are variations in the definitions of variables, many missing data and a limited number of variables that can be collected when medical records are used.

There were differences in defining and categorising the delay. Most studies divided delays into patient and system delays [8,13], and many studies used a cut off point of three months for categorising delay [5,8,9,14,20]. In our study, we use a six-month cut off point for delay because our respondents experienced longer delay.

There was a selection bias in recruiting respondents because they were included after the diagnosis of breast cancer was made. Our study did not include those who had died before the study was conducted or those who were lost to follow up. However, it was assumed that those who died early had an advanced stage of disease and had longer delay, suggesting that the diagnosis delay was under-estimated in our study.

Selection bias also occurred because our study was conducted in hospitals and some patients may not have presented to the hospital at all, instead preferring alternative treatment. A population-based study was not possible because of logistic problems in enrolling those patients.

Furthermore, there was information bias in this study. Our study method relied on the patients' recall of the events leading up to their diagnosis. Patients who delayed consultation and diagnosis needed to remember more distant events than those who did not delay. We interviewed respondents after the diagnosis was made instead of after the recognition of symptoms, so some of the variables could not be measured retrospectively. We also could not obtain detailed information on the type and quality of the diagnostic procedures because we were not involved in the management of the patients and procedural details were not included in the patients' medical records.

## Conclusions

Our study found that consultation and diagnosis delays are very serious problems in Malaysia. The factors significantly associated with diagnosis delay were the use of alternative therapy, breast ulcer, palpable axillary lymph nodes, false-negative diagnostic test, non-cancer interpretation and a negative attitude toward treatment. Diagnosis delay was influenced by a complex interaction of many factors. Breast awareness and education are needed to reduce breast cancer mortality by promoting early detection, diagnosis and treatment before the tumour enlarges and spreads to lymph nodes and metastasis. Women should be educated that breast cancer does not always present as a painless lump; it can also present with pain, dimpling, swelling or nipple discharge. We advocate self and clinical breast examinations for women because the practice is still not at an optimum level in Malaysia [31], even though there are reports in the literature indicating that breast self examination failed to reduce breast cancer mortality [32]. The Ministry of Health and non-governmental organisations need to increase and widen their coverage and target groups. Furthermore, clinical practice guidelines for breast cancer management should incorporate time guidelines for diagnosis and treatment.

## Competing interests

The authors declare that they have no competing interests.

## Authors' contributions

BN participated in the conception and design of the study, obtaining of funding, acquisition, analysis and interpretation of the data and drafting the manuscript. KGR participated in the conception and design of the study, obtaining of funding and revising the manuscript critically. MAR participated in the conception and design of the study, analysis and interpretation of data and drafting the manuscript. NNN participated in the obtaining of funding, analysis and interpretation of data. BMB participated in the enrolment of patients and acquisition of data. All authors read and approved the final manuscript.

## Pre-publication history

The pre-publication history for this paper can be accessed here:

http://www.biomedcentral.com/1471-2407/11/141/prepub
